# Intelligent Trajectory Tracking Behavior of a Multi-Joint Robotic Arm via Genetic–Swarm Optimization for the Inverse Kinematic Solution

**DOI:** 10.3390/s21093171

**Published:** 2021-05-03

**Authors:** Mohammad Soleimani Amiri, Rizauddin Ramli

**Affiliations:** Department of Mechanical and Manufacturing Engineering, Faculty of Engineering and Built Environment, Universiti Kebangsaan Malaysia, Bangi 43600, Selangor, Malaysia; msa0911@gmail.com

**Keywords:** robotic arm, Genetic Algorithm, Particle Swarm Optimization, PID control

## Abstract

It is necessary to control the movement of a complex multi-joint structure such as a robotic arm in order to reach a target position accurately in various applications. In this paper, a hybrid optimal Genetic–Swarm solution for the Inverse Kinematic (IK) solution of a robotic arm is presented. Each joint is controlled by Proportional–Integral–Derivative (PID) controller optimized with the Genetic Algorithm (GA) and Particle Swarm Optimization (PSO), called Genetic–Swarm Optimization (GSO). GSO solves the IK of each joint while the dynamic model is determined by the Lagrangian. The tuning of the PID is defined as an optimization problem and is solved by PSO for the simulated model in a virtual environment. A Graphical User Interface has been developed as a front-end application. Based on the combination of hybrid optimal GSO and PID control, it is ascertained that the system works efficiently. Finally, we compare the hybrid optimal GSO with conventional optimization methods by statistic analysis.

## 1. Introduction

With the advancements in robotic technology, numerous types of robots have become involved in our daily life and help humans in many different areas. As one of the most common robots, the multi-joint manipulator robotic arm plays an important role in automotive, agriculture and bio-medical sectors due to its flexibility, robustness and accuracy [1,2,3].

The identification of the Inverse Kinematic (IK) plays an important role in the precision control of trajectory tracking [4,5]. Various IK solutions have been carried out for robotic arms [6,7]. For instance, Xu et al. [8] presented a combination brain of a computer interface and computer vision to move a robotic arm end-effector to a desired point by using a depth camera. A six degree of freedom (6DoF) robot with initialized commands from a user’s brain signals combined with a point clouds model was verified with five healthy candidates without specific user training, showing acceptable accomplishment for complex tasks. Fang et al. [9] established a visual communication method using deep neural networks, in which the movements of a human arm were monitored and determined by the Denavit–Hartenberg (D-H) technique. Narayan et al. [10] presented a 5DoF robotic arm with a three-finger gripper and validated the IK in a simulation platform. Ye et al. [11] dealt with 5DoF manipulator forward-IK problems using Ferrari’s and redundant Euler methods and validated them in a simulation model. Wei at al. [12] applied a neural network to a robotic arm and used environment feedback to reach a specific target point inspired by animal and human biological neural networks. They validated their approach using the penalty function to avoid the robot from reaching specific points. The results show the end-effector reached the target successfully. Ren et al. [13] developed generative neural networks to solve the IK for a robotic arm. They determined the IK by the D-H technique and Moveit, which is an application of a Robot Operating System (ROS) to control and monitor a robot.

Traditionally, the IK has been utilized to establish joint configurations of manipulators based on the end-effector position. However, the traditional IK methods cannot consider the continuity of configurations, collision avoidance and kinematic singularities that arises when attempting to follow the end-effector path [14]. In addition, solving IK problems is a difficult challenge because manipulators with more than 5DoF result in an infinite number of possible solutions for joint trajectories that determine the same position in the Cartesian space [15]. Traditional analytical solutions cannot directly calculate the one-to-many possible relationships in the Cartesian space. Therefore, evolutionary algorithms such as optimization methods are used to solve IK problems quantitatively [11,16]. For instance, Starke et al. [17] studied a mimetic evolutionary algorithm, which was a combination of the Genetic Algorithm (GA), Particle Swarm Optimization (PSO) and gradient-based optimization to address the IK solution for various industrial and anthropomorphic robots.

One of the approaches used in this paper is to combine GA and PSO in order to develop an optimal solution for the IK and Proportional–Integral–Derivative controller (PID) controller tuning. This optimization method has been presented in several works for various applications [18,19,20]. For instance, Dziwinski, et al. [21] presented a fuzzy-logic controller in which a combination of PSO and GA was used in parallel to improve PSO performance by adding crossover and mutation to the GA to avoid becoming trapped in local optima. Farand et al. [22] developed a combination of GA and PSO to reduce computational time and accuracy in comparison with other known methods such as GA and PSO for high-dimensional and complex functions.

The PID controller is one of the most common used classical control systems in different industries because of its flexibility, satisfactory results [23,24], ease of implementation in a control system and wide usage in industries [25,26]. In order to enhance the accuracy and robustness of classical PID control, one of the techniques is to combine it with optimization methods [27,28]. Belkadi et al. [29] worked on PSO with a random initial value to tune the parameters of the PID controller by minimizing the trajectory error. They verified their controller in a simulation model and compared it with conventional methods by numerical analysis. Phu et al. [30] used optimization with sliding mode control based on the Bolza–Meyer criterion to minimize the vibration effect. In another work, Suhaimin et al. [31] used a PID controller for a 5DoF robotic arm and controlled its joints for the point-to-point trajectory tracking of end-effectors.

The contribution of this paper is the development of an optimal hybrid IK and PID controller for joint trajectory tracking, using the Genetic–Swarm Optimization technique. The applicability of the proposed technique for the IK solution of end-effectors and steady-state error for control is compared with conventional optimization approaches such as the GA and PSO. In addition, a 5DoF robotic arm is selected for analysis due to its simple structure, flexible action, small volume, convenient operation and so on; this device is widely used in many fields and industries [32].

The rest of the paper is organized as follows: first, the kinematic and dynamic models of the 5DoF robotic arm are established using the D-H and Lagrangian method. Subsequently, an IK solution is determined by hybrid Genetic–Swarm Optimization (GSO) for angular trajectories of each joint reaching the target position. The joint angles determined by the IK are implemented in a closed-loop system using a PID controller, and the gains are tuned by the GA and PSO. The 3D models of the robotic arm are simulated in the Gazebo environment. Finally, a Graphical User Interface (GUI) is created to interact with the 3D model in the ROS environment.

## 2. Dynamic and Kinematic Model

**Figure 1:**
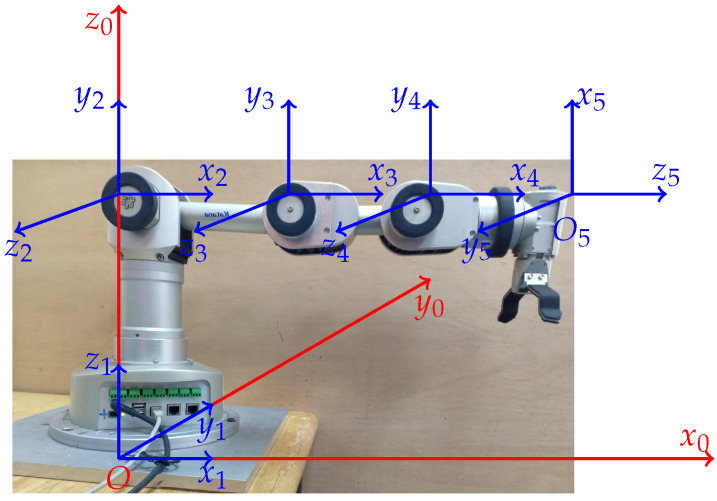
Configuration of the robotic arm, where $x_{0}$, $y_{0}$ and $z_{0}$ are axes of the reference frame.

The robotic arm consists of a base, four links, a wrist and gripper that are connected to each other by joints in series. Figure 1 represents a 5DoF robotic arm, in which the coordinate systems of joints and global frames are presented.

There are various methods used to determine the dynamic equation of robot manipulators, such as Newton–Euler, Kane and Hamilton approaches. In this work, an energy-based Lagrangian method has been adopted to determine the relation between the torque and angle of joints; one of the advantages of the Lagrangian method is that, unlike the Newton–Euler method, it is not necessary to determine internal forces between joints; therefore, it is quicker and easier to obtain the equation of motion [33]. The Lagrangian equation is given as follows:

(1) $$L=E_{k}-E_{p}$$

(2) $$\tau_{i}=\frac{d}{dt}\left(\frac{\partial L}{\partial \dot{\theta_{i}}}\right)-\left(\frac{\partial L}{\partial \theta_{i}}\right)+B_{i}\left({\dot{\theta }}_{i}\right)$$

where $B_{i}$ is the joint friction coefficient; *L* is the Lagrangian function; $T_{i}$ is the torque of each link, with i=1,2,3,4,5; $\theta_{i}$ and $\dot{\theta_{i}}$ are the angular trajectory and velocity; and $E_{p}$ and $E_{k}$ are the total potential and kinetic energies, respectively. From [34], the equations of $E_{p}$ and $E_{k}$ are determined as follows:

(3) $$E_{p}=\sum_{i=1}^{5}m_{i}gz_{di}$$

(4) $$E_{k}=\sum_{i=1}^{5}\left[\frac{1}{2}m_{i}({\dot{x}}_{di}^{2}+{\dot{y}}_{di}^{2}+{\dot{z}}_{di}^{2}\right)+\frac{1}{2}I_{xi}{\dot{\theta }}_{i}^{2}+\frac{1}{2}I_{i}]$$

where $m_{i}$ and $I_{i}$ are the mass and inertia of each link; *g* is the gravity acceleration; and $\left({\dot{x}}_{di},{\dot{y}}_{di},{\dot{z}}_{di}\right)$ is the time derivative of the centroid position of each joint, where i=1,2,3,4,5. According to the geometric relation, the centroid position $\left(x_{di},y_{di},z_{di}\right)$ of every linkage is written as

(5) $$X_{d_{i}}=\sum_{j=1}^{i-1}\left(R_{z_{j}}.^{j}X\right)+R_{z_{i}}.^{i}X_{d}$$

where ${}^{j}X\in {ℜ}^{3\times 1}$ is the position of joint ${\left(i-1\right)}_{th}$ according to the reference frame; $X_{d_{i}}\in {ℜ}^{3\times 1}$ is the position of the centroid point of link $i_{th}$ relative to the reference frame; $R_{z_{i}}\in {ℜ}^{3}$ is the rotation matrix around z-axes according to the coordinate system placed in the $i_{th}$ joint; and ${}^{i}X_{d}\in {ℜ}^{3\times 1}$ represents the centroid position of the link $i_{th}$ regarding the coordinate system located in the joint $i_{th}$. By substituting $E_{k}$ and $E_{p}$ in the Lagrangian function, the state space dynamic is determined in range of motion condition where while one joint is moving, the other ones are fixed, which is shown as follows:

(6) $$\tau =M\ddot{\theta }+V\dot{\theta }+G\left(\theta \right)$$

where $\theta \in {ℜ}^{5\times 1}$ and $\ddot{\theta }\in {ℜ}^{5\times 1}$ are the angular rotation and acceleration; $\tau \in {ℜ}^{5\times 1}$ is the torque vector; and $M\in {ℜ}^{5}$ is a matrix containing mass and inertia elements, which is shown as follows:

(7) $$M=\left[\begin{array}{ccccc} e_{m_{1}} & 0 & 0 & 0 & 0\\ 0 & e_{m_{2}} & 0 & 0 & 0\\ 0 & 0 & e_{m_{3}} & 0 & 0\\ 0 & 0 & 0 & e_{m_{4}} & 0\\ 0 & 0 & 0 & 0 & e_{m_{5}} \end{array}\right]$$

where $e_{m_{i}}\;i=1,2,3,4,5$ represents the mass and inertia elements, expressed as follows:

(8) $$e_{m_{1}}=I_{x1};\;e_{m_{i}}=l_{i}^{2}\sum_{i=i+1}^{5}\left(m_{i}\right)+I_{i}+m_{i}l_{c_{i}}^{2}$$

where $l_{c_{i}}$ is the length of the centroid position for each link and $l_{i}$ is the length of every link. $V\in {ℜ}^{5}$ is the centrifugal, coriolis and friction matrix and $G\left(\theta \right)\in {ℜ}^{5}$ represents the gravity matrix, expressed as follows:

(9) $$V\left(\dot{\theta },\theta \right)=B_{i}\cdot I_{5\times 5}\;G\left(\theta \right)=e_{g_{i}}\cdot I_{5\times 5}$$

where $I_{5}\in {ℜ}^{5}$ is the identity matrix and $e_{g_{i}}$ shows the elements of mass and gravity matrices, represented as follows:

(10) $$e_{g_{i}}=\left(l_{i}\sum_{i=i+i}^{5}\left(m_{i}\right)+l_{c_{i}}m_{i}\right)gsin\left(\theta_{i}\right)$$

where *g* represents gravitational acceleration. Table 1 illustrates the physical features of the robotic arm’s links.

In the next stage, the forward kinematic based on modified D-H (mD-H) algorithms has been developed to establish the relative position of the 5DoF robotic arm end-effector to its reference frame *O* [35]. Table 2 represents the parameters of the mD-H.

In Table 2, $\alpha_{i-1}$, $\theta_{i}$, $d_{i}$ and $a_{i-1}$ represent the twist angle, joint angle, link offset and link length, respectively. The mD-H homogeneous transformation is expressed as follows:

(11) $${}_{i}^{i-1}T=\left[\begin{array}{cccc} cos\theta_{i} & -sin\theta_{i} & 0 & a_{i-1}\\ sin\theta_{i}cos\theta_{i-1} & cos\theta_{i}cos\theta_{i-1} & -sin\alpha_{i-1} & -d_{i}sin\alpha_{i-1}\\ sin\theta_{i}sin\alpha_{i-1} & cos\theta_{i}sin\alpha_{i-1} & cos\alpha_{i-1} & d_{i}cos\alpha_{i-1}\\ 0 & 0 & 0 & 1 \end{array}\right]$$

The transformation matrix of the end-effector is the transformation matrix from the reference frame to the last frame, which is shown as follows:

(12) $${}_{5}^{0}T{=}_{1}^{0}T\cdot_{2}^{1}T\cdot_{3}^{2}T\cdot_{4}^{3}T\cdot_{5}^{4}T$$

where ${}_{1}^{0}T$, ${}_{2}^{1}T$, ${}_{3}^{2}T$, ${}_{4}^{3}T$ and ${}_{5}^{4}T$ are the transformation matrices of each joint to its previous joint. The transformation matrix from the reference frame to end-effector is as follows:

(13) $${}_{5}^{0}T=\left[\begin{array}{cccc} t_{1,1} & t_{1,2} & t_{1,3} & t_{1,4}\\ t_{2,1} & t_{2,2} & t_{2,3} & t_{2,4}\\ t_{3,1} & t_{3,2} & t_{3,3} & t_{3,4}\\ t_{4,1} & t_{4,2} & t_{4,3} & t_{4,4} \end{array}\right]$$

where $t_{1,4}$, $t_{2,4}$ and $t_{3,4}$ express the end-effector position relative to the reference frame, which is shown as follows:

$$x=t_{1,4}=-\frac{1}{2}(l_{4}sin\left(\theta_{4}+\theta_{3}+\theta_{2}+\theta_{1}\right)-l_{4}\sin\left(\theta_{4}+\theta_{3}+\theta_{2}-\theta_{1}\right)+l_{3}cos\left(\theta_{3}+\theta_{2}+\theta_{1}\right)+$$

(14) $$l_{3}cos\left(\theta_{3}+\theta_{2}-\theta_{1}\right)+l_{2}cos\left(\theta_{2}+\theta_{1}\right)+l_{2}cos\left(\theta_{2}-\theta_{1}\right))$$

$$y=t_{2,4}=\frac{1}{2}(l_{4}cos\left(\theta_{4}+\theta_{3}+\theta_{2}+\theta_{1}\right)-l_{4}cos\left(\theta_{4}+\theta_{3}+\theta_{2}-\theta_{1}\right)+l_{3}sin\left(\theta_{3}+\theta_{2}+\theta_{1}\right)-$$

(15) $$l_{3}sin\left(\theta_{3}+\theta_{2}-\theta_{1}\right)+l_{2}sin\left(\theta_{2}+\theta_{1}\right)-l_{2}sin\left(\theta_{2}-\theta_{1}\right))$$

(16) $$z=t_{3,4}=l_{4}cos\left(\theta_{4}+\theta_{3}+\theta_{2}\right)+l_{3}sin\left(\theta_{3}+\theta_{2}\right)+l_{2}sin\left(\theta_{2}\right)$$

## 3. Optimal Inverse Kinematic

Since the number of variables is greater than the number of equations and the end-effector position is non-linear, the usage of traditional methods such as Gaussian elimination are not practical [36]. Thus, in this paper, the IK is defined as a mono-objective optimization problem. The desired position of the end-effector is set to be achieved by minimizing the objective function. In this study, the hybrid version of the GA and PSO, named GSO, is adopted to solve the IK problem, because GA is developed initially by random values due to its reliability and robust performance [37] and PSO is sufficient to find accurate results in a few iterations with low computational time. Subsequently, PSO is initialized by the results of the GA. In the GSO algorithm, the GA provides searching space and initial values for PSO to avoid becoming trapped in local optima.

**Figure 2:**
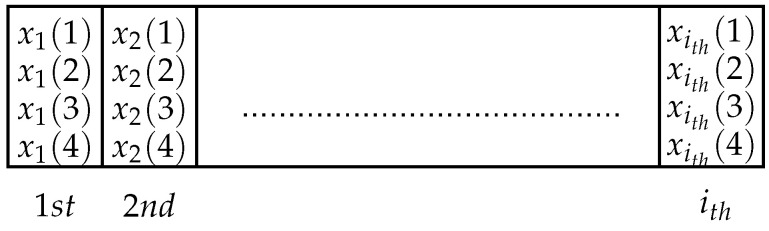
Structure of population for an iteration.

The summation of squared error (SSE), which is a well-known statistic in multiple regression analyses [38], is chosen as an objective function because it shows the squared sum of residuals, which is the error between the measured and desired trajectory of the end-effector, and illustrates how close a regression line is to a set of residuals. The squaring is necessary to remove any negative signs. The objective function is given as follows:

(17) $$f_{obj}=\sqrt{{\left(e_{x}\right)}^{2}+{\left(e_{y}\right)}^{2}+{\left(e_{z}\right)}^{2}}$$

where $e_{x}$, $e_{y}$ and $e_{z}$ are the errors, represented as follows:

(18) $$e_{x}=x-x_{des}$$

(19) $$e_{y}=y-y_{des}$$

(20) $$e_{z}=z-z_{des}$$

where $x_{des}$, $y_{des}$ and $z_{des}$ are the desired positions of the endpoint regarding the reference frame. *x*, *y* and *z* express the position of the endpoint, which are determined by the gene of the GA from Equations (18)–(20). The population structure of an iteration is represented in Figure 2.

In each population, there is a gene which consists of each joint angle of the robotic arm, represented as $x_{1}$, $x_{2}$, $x_{3}$ and $x_{4}$, which are $\theta_{1}$, $\theta_{2}$, $\theta_{3}$ and $\theta_{4}$, respectively. In the robotic arm model, there are limitations for the angular trajectory of each joint, which create the searching space for the GA, which is as follows:

(21) $$-3.02\left(rad\right)\leq \theta_{1}\leq 2.89\left(rad\right)$$

(22) $$-0.13\left(rad\right)\leq \theta_{2}\leq 2.16\left(rad\right)$$

(23) $$-2.22\left(rad\right)\leq \theta_{3}\leq 2.05\left(rad\right)$$

(24) $$-2.03\left(rad\right)\leq \theta_{4}\leq 1.87\left(rad\right)$$

(25) $$\theta_{5}=1.57\left(rad\right)$$

$\theta_{5}$ is set as 90 degrees and is not included in the design variables because it is assumed that the gripper is located at last link point down to grab the objects. The first iterations of the GA are set randomly within the searching space, as demonstrated in Equations (21)–(24). After the initialization, the objective function is calculated for each gene of the population for evaluation and sorted in ascending order. The next iterations are created by crossover and mutation. The crossover enhances the possibilities of finding the most optimum results by blending the previous iterations as children and parents using the uniform crossover operator. In addition, mutation is performed to maintain the diversity of the GA [39,40,41]. The algorithm is continued by the evaluation of each gene by determining the objective function followed by sorting in ascending order. This trend continues until the maximum iterations are reached. In the last iteration, because of the ascending sorting, the first gene is the result of GA and represents the optimum angles of joints which are needed to lead the endpoint to reach the desired position.

(26) $$x_{ga}=\left[\theta_{1},\theta_{2},\theta_{3},\theta_{4}\right]$$

$x_{ga}$ is the output of GA which is used to create the range for the initial population of the PSO, which is shown as follows:

(27) $$x_{1,j}=rand\left[x_{min},x_{max}\right]$$

where *j* stands for the number of particles in the first population and rand is the function used to generate a random value between $x_{min}$ and $x_{max}$, which are the lower and higher bounds, given as follows [42]:

(28) $$x_{min}=x_{ga}-r$$

(29) $$x_{max}=x_{ga}+r$$

where $r\in {ℜ}^{1\times 4}$ is a random vector between zero and one. After creating the particles of the first population, the objective function is determined for each particle to evaluate and sort in descending order. The particles of population for the next iteration are created as follows:

(30) $$x_{i+1,j}=x_{i,j}+v_{i+1,j}$$

where $x_{i+1,j}$ is the particle of the next iteration. $v_{i+1,j}\in {ℜ}^{1\times 4}$ is a vector that represents the velocity and direction of each particle through the particle of the next iteration, which is shown as follows:

(31) $$v_{i+1,j}=\omega_{i}v_{i,j}+c_{1}r\left(p_{best}-x_{i,j}\right)-c_{2}r\left(g_{best}-x_{i,j}\right)$$

where $p_{best,i}$ is called the best position, containing the particles that have the minimum objective function. $g_{best}$ is the global best, including the particles which are the minimum of the $p_{best}$, which is shown as follows:

(32) $$g_{best}=min\left\{p_{best_{i}}\right\}\;i=1,2,\ldots ,i_{max}$$

where *i* and $i_{max}$ are the current and maximum number of iterations, respectively. In the first iteration, after evaluation, the minimum particle is saved as $p_{best}$ and $g_{best}$, and the velocity is a zero vector.

(33) $$v_{1,j}=\left[0,0,0,0\right]$$

In Equation (31), $\omega_{i}$ is the inertia weight, where its adjustable value for each iteration is given by the following equation:

(34) $$\omega_{i+1}=\omega_{damp}\omega_{i}$$

where $\omega_{damp}$ is the damping value for ω, set as 0.05, and $c_{1}$ and $c_{2}$ are coefficients of self and social recognition, respectively. The value of $c_{1}$ is greater than $c_{2}$, and their summation should remain at 4 in all iterations [43].

(35) $$c_{1}=1.8b+2.1$$

(36) $$c_{2}=1.8a+0.1$$

where, *a* and *b* are the ascending and descending gains between zero and one, represented as follows:

(37) $$a=\frac{i}{i_{max}}\;i=1,2,\ldots ,i_{max}$$

(38) b=1−a

**Figure 3:**
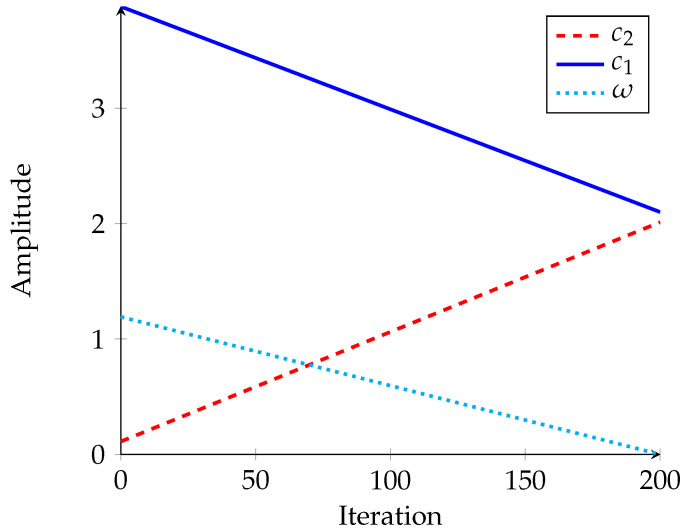
Changes in parameters of modified PSO.

Figure 3 represents the changes of parameters of PSO during all iterations. The initial values for $c_{1}$, $c_{2}$ and ω are 3.9, 0.1 and 1.2, respectively [44].

**Figure 4:**
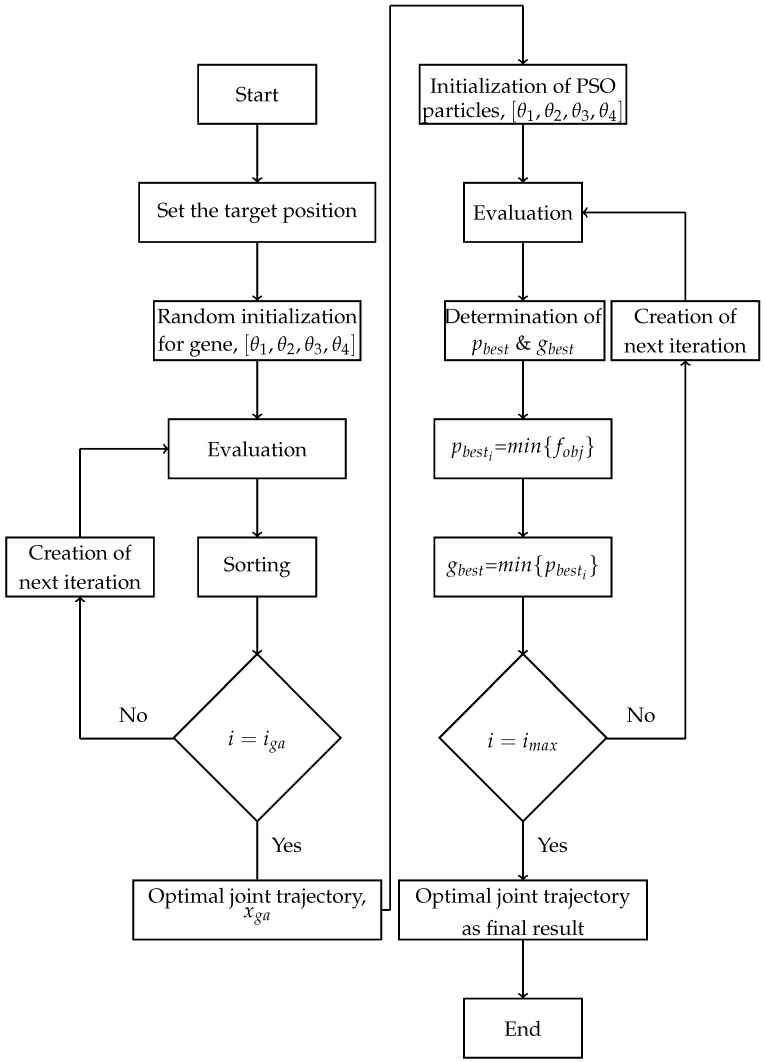
Flow chart of GSO.

After generating each population, the evaluation and sorting of its particles is developed. This trend is followed until the number of iterations is equal to $i_{max}$. In this paper, the size of the population for GA and PSO is 40 and $i_{max}$ is 200 for each. Algorithm 1 and Figure 4 show the pseudo-code and flow chart of GSO.
**Algorithm 1** Pseudo code of GSO 1:Start; 2:  3:Set the target position of the endpoint; 4:  5:Start GA; 6:  7:Initialize the first population randomly; 8:  9:Evaluate initial population;10: 11:**while** Number of iterations equal to maximum iteration of GA **do**;12: 13:    Create new iteration using crossover and mutation;14: 15:    Evaluate the population by determining the objective function;16: 17:    Sort the genes in ascending order;18: 19:**end while**20: 21:Select the first gene of the last iteration as the result;22: 23:End GA;24: 25:Start PSO;26: 27:Initialize particles of the first population of PSO based on GA results;28: 29:Evaluate the first population;30: 31:**while** Number of iterations equal to maximum iteration of PSO **do**;32: 33:    Create new population;34: 35:    Evaluate the particles of population;36: 37:    Set the minimum particle as the $P_{best}$;38: 39:    Set the minimum $P_{best}$ as $g_{best}$;40: 41:**end while**42: 43:Establish particle of the $g_{best}$ as the results;44: 45:End.

## 4. Control System and Tuning

**Figure 5:**
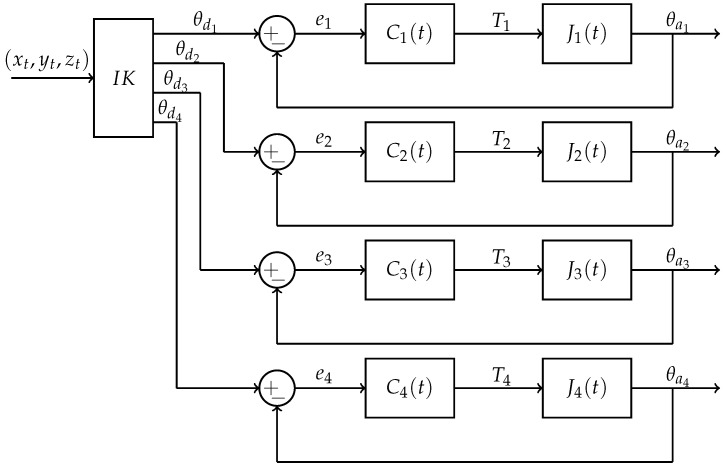
Block diagram of control system for each joint.

A closed-loop control system is developed for each joint, and its parameters are adjusted by GSO to converge by adjusting the required torque toward each joint. The desired angular trajectory is determined by the IK. Figure 5 demonstrates the control system of the robotic arm.

In Figure 5, $J_{1}\left(t\right)$, $J_{2}\left(t\right)$, $J_{3}\left(t\right)$ and $J_{4}\left(t\right)$ are plants of each joint; $\left(x_{t},y_{t},z_{t}\right)$ is the desired position; $\theta_{d_{1}}$, $\theta_{d_{2}}$, $\theta_{d_{3}}$ and $\theta_{d_{4}}$ are the desired angular trajectory determined by the IK; $\theta_{a_{1}}$, $\theta_{a_{2}}$, $\theta_{a_{3}}$ and $\theta_{a_{4}}$ are the actual angular trajectory for each joint; and $e_{1}$, $e_{2}$, $e_{3}$ and $e_{4}$ are the trajectory errors that are the difference between the desired and actual angular trajectory, given as follows:

(39) $$e_{i}=\theta_{d_{i}}-\theta_{a_{i}}\;i=1,2,3,4$$

The PID controllers $C_{1}\left(s\right)$-$C_{4}\left(s\right)$ for each joint are given as follows:

(40) $$C_{i}\left(t\right)=K_{P}e_{i}\left(t\right)+K_{I}\int e_{i}dt+K_{D}\frac{de_{i}}{dt}$$

The tuning of the PID controller is assumed to be an optimization problem, and its parameters are defined as design variables. The tuning processes are carried out by the GSO algorithm, in which the GA starts to optimize the design variables based on random initial parameters, and subsequently the algorithm is continued by PSO based on the output of the GA. Initial parameters of the first population are randomly chosen between 0 and 1, given as follows:

(41) $$x_{1,j}=rand\left[0,1\right]\;j=1,...,j_{max}$$

where *x* is the particle of the PSO and gene of the GA in each population and *j* and $j_{max}$ are the current and maximum number of populations. After setting the initial values for particles, an evaluation is carried out based on the objective function of tuning, which is the absolute steady-state error:

(42) $$f=|\theta_{act}-\theta_{des}|$$

where $\theta_{act}$ and $\theta_{des}$ are the actual and desired joint trajectory, respectively. $\theta_{act}$ is measured from a simulation model in real-time, and $\theta_{des}$ is developed by the optimal IK. After evaluation and sorting in descending order, the particles of the population for next iteration are generated by mutation and crossover. Whenever the number of iterations meets half of the maximum iterations, the algorithm is switched to PSO. The searching space of PSO is limited around the results of the GA to lead the algorithm toward global optima, as follows:

(43) $$x_{i,j}=\left[x_{ga}-\frac{x_{ga}}{2},x_{ga}+\frac{x_{ga}}{2}\right]$$

The next populations of PSO are created by the particles of the next iteration. The algorithm continues until the number of iterations reaches the maximum. The output is an optimal set for PID parameters.

## 5. Results and Discussion

**Figure 6:**
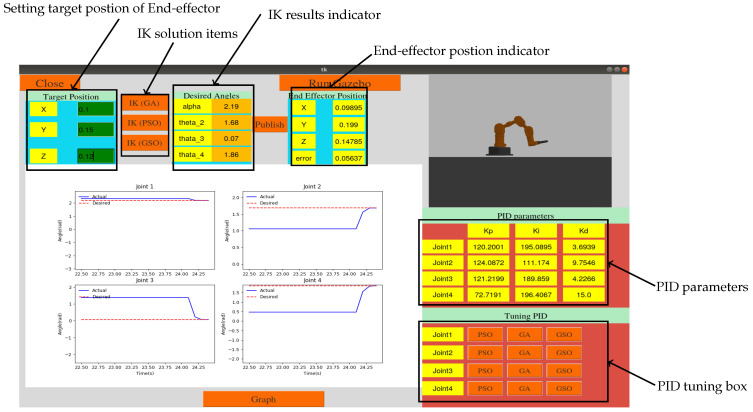
GUI for simulation model.

The optimal IK and PSO tuning of controllers was applied in the 3D simulation of a robotic arm in a 3D environment to simulate robots integrated with ROS [45]. A GUI was programmed by using Python to run the algorithms and communicate with the simulation model. In addition, by providing a camera in the Gazebo environment, it was possible to monitor the results visually and numerically, as shown in Figure 6.

The desired position for the three different algorithms—i.e., GA, PSO and GSO—could be selected according to the IK method, and the actual position of end-effector, error of the actual trajectory and desired trajectory of each joint could be monitored. In addition, the controller parameters could be tuned in real time and observed. Table 3 compares the optimal results for GA, PSO and GSO for the IK solution, while $f_{obj}$ is the SSE for various sets of optimization parameters.

Various sets of parameters were defined to observe the influences of changes in parameters on the optimization algorithms.

- For GA, $set_{ga}^{1}$: crossover = 0.9, mutation = 0.1, population = 40 and generation = 400; $set_{ga}^{2}$: crossover = 0.8, mutation = 0.2, population = 40, and generation = 400; $set_{ga}^{3}$: crossover = 0.7, mutation = 0.3, population = 40 and generation = 400;
- For PSO, $set_{pso}^{1}$: particles = 20, and generation = 200; $set_{pso}^{2}$: particles = 30 and generation = 300; $set_{pso}^{3}$: particles = 40 and generation = 400;
- For GSO, $set_{gso}^{1}$: crossover = 0.9, mutation = 0.1, population of GA and particles of PSO = 40, generation of GA = 300 iteration of PSO = 100, $set_{gso}^{2}$: crossover = 0.8, mutation = 0.2, population of GA and particles of PSO = 40, generation of GA = 200 iteration of PSO = 200, $set_{gso}^{3}$: crossover = 0.7, mutation = 0.3, population of GA and particles of PSO = 40, generation of GA = 100 and iteration of PSO = 300.

The mean of $f_{obj}$ for GSO in $set_{gso}^{3}$ has the lowest $f_{obj}$ of 7.9$\times 10^{-15}$, and the maximum of $f_{obj}$ is 4.99$\times 10^{-14}$, which is the nearest value to its mean compared to other results. This causes the lowest variance of all tests. The mean of the PSO results is lower than GA, while GSO shows the minimum results, which represents a significant improvement for the results obtained by the GSO algorithm. This is due to the hybrid of the GA and PSO algorithms in series; creating the initial values of PSO based on results of the GA increases accuracy compared to using each algorithm individually. In addition, by increasing the number of iterations and particles of PSO, GSO and PSO algorithms show improvements in their results.

The H-values were measured by the Kruskal–Wallis method and were 0.03, 16.07 and 15.84 for the GA, PSO and GSO respectively. The test was calculated with the assumption of α=0.05; therefore, the critical value for this test was 5.99. Since PSO and GSO had greater H-values than critical points, there were significant differences among the groups of $f_{obg}$ calculated by PSO and GSO.

Table 4 represents the computational time in seconds for GA, PSO and GSO, while the parameters are determined as $set_{ga}^{3}$, $set_{pso}^{3}$ and $set_{gso}^{3}$, respectively.

The mean computational time of PSO was less than GA and GSO by 5.82 s and 1.37 s, respectively. Although the computational time of PSO was the lowest, the combination of GA and PSO reduced the computational time consumption significantly compared to GA by 4.45 s. By considering the value of $f_{obj}$ of GSO in Table 3 and the computational time, the usage of GSO for IK solution can be seen to have resulted in significant improvements in accuracy and computational time consumption.

In order to test the IK results solved by GSO in the robotic arm model in the Gazebo environment, nine desired position coordinates were expressed. Table 5 illustrates the coordinates of the nine target positions and the angles for each joint.

**Figure 7:**
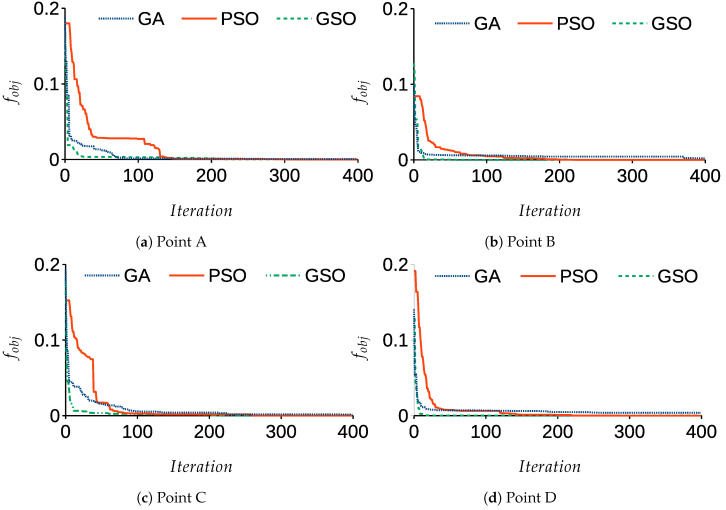
The objective functions over iterations.

Figure 7 shows the objective function $f_{obj}$ for three ways of tuning PID parameters with 400 iterations, and the parameters of GSO were the same as $set_{gso}^{3}$ in Table 3.

From the results, it can be observed that GSO converges faster than GA and PSO, because by establishing the angle of joints for the desired points, the angular trajectories are exported to the control system and then tuned by GSO. The performance of the closed-loop control system is validated for each joint, in which the angular trajectories solved by the IK are set as desired ($\theta_{d_{i}}$). Table 6 represents the tuned PID parameters.

**Figure 8:**
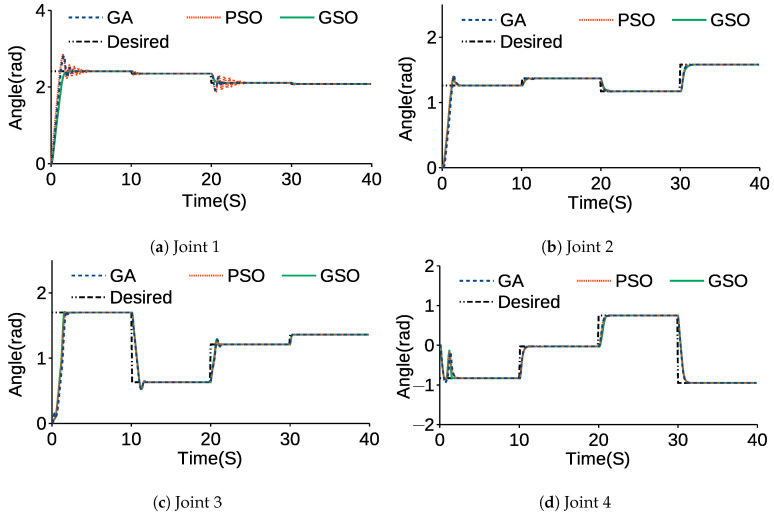
Angular trajectory of each joint.

**Figure 9:**
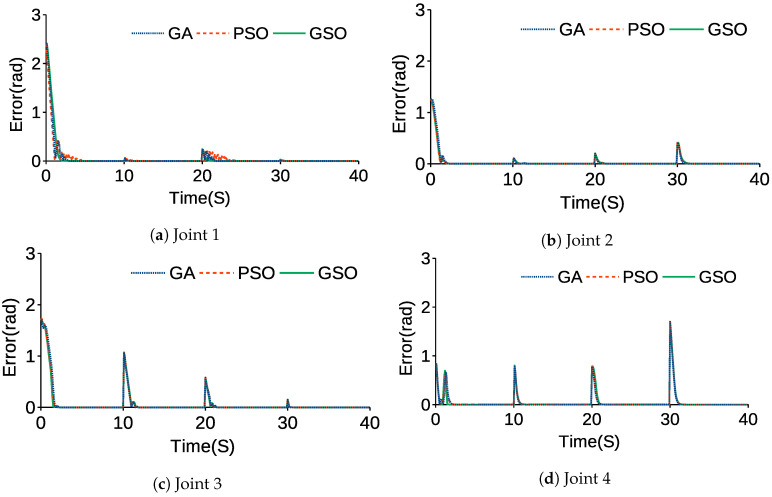
Angular trajectory error of each joint.

**Figure 10:**
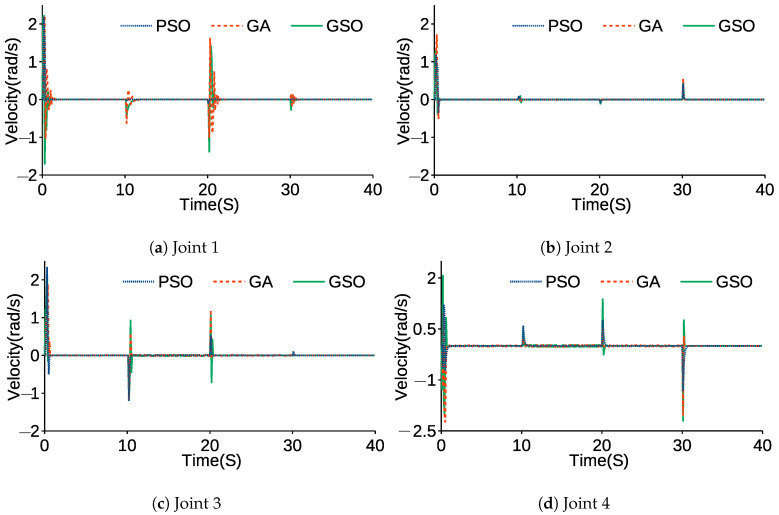
Angular velocity of each joint.

Figure 8 and Table 7 compare the angular trajectories and average errors tuned by the GA, PSO and GSO while the end-effector moved to the desired positions and its PID parameters were tuned by the GA, PSO and GSO. Figure 9 and Figure 10 show the error and angular velocity for each joint while the end-effector tracked points A, B, C, and D.

In Figure 8 and Figure 9, overshoot can be observed when the joints are changing trajectories. In Figure 10, there are fluctuations when there is a change in position of the joints and they are tracking each point. The rest of the graph levels off at zero. This shows the stability of the control system, because while joints do not move and are in a stable situation, the joints do not shake. In Table 7, $AE_{GA}$, $AE_{PSO}$ and $AE_{GSO}$ are averages of the SSE for GA, PSO and GSO. The actual trajectory of each joint shows the significant effects of GSO compared to PSO and the GA due to the initialization of PSO by results of the GA for GSO, which allows the algorithm to find the global optima more accurately. From $AE_{GA}$, $AE_{PSO}$ and $AE_{GSO}$, it can be concluded that the GSO resulted in a lower value because of its efficiency in finding precise results and lower chance of becoming trapped in local optima.

## 6. Conclusions

This paper presented a hybrid optimal IK solution of a 5DoF robotic arm to determine the joint trajectories based on its end-effector position. The Denavit–Hartenberg method was used to establish the kinematic and was solved by GSO, which is a combination of the GA and PSO. The trajectories were implemented with PID control as desired trajectories for each joint. The tuning of PID parameters was presented as optimization problem and carried out by GSO. A GUI was created to operate and visualize the performance of the robotic arm in a virtual environment. This method addresses the issue of finding one-to-many possible solutions of IK for a 5DoF robotic arm to control each joint efficiently and precisely to determine end-effector positions in Cartesian space.

The results show that GSO has a lower average error for each joint than PSO and GA. For instance, for joint 4, the average error for one specific path for GSO is 19.33% and 22.7% less than PSO and the GA, respectively. These results show that initialization particles for PSO by using the GA can give more accurate results and avoid algorithms becoming trapped in local optima. In addition, the mean computational time for GSO is lower than the GA by 4.45 s and higher than PSO by 1.37 s. Therefore, GSO is a sufficient algorithm for IK solution for robotic arms.

This method can be used for any robotic arms to control their end-effector. The limitation of this work is that we did not apply the hybrid proposed method to a real robotic arm. In addition, the target position of the end-effector was chosen by the user. Therefore, in future work, this position can be issued by sensors such as depth camera or tag marker measurement algorithms. Furthermore, the proposed IK and control system developed by the GSO algorithm was validated in the 3D simulation environment of Gazebo, and the effect of sensor noises was not considered; this can be covered in the future work. In addition, the proposed method can be tested for applications in which the accurate position of end-effectors is needed, such as welding, material handling and thermal spraying or any other industrial applications.

## Figures and Tables

**Table 1:** Physical features of the robotic arm.

link	$l_{i}\left(m\right)$	$l_{c_{i}}\left(m\right)$	$m_{i}\left(\mathrm{Kg}\right)$	$I_{i}$	$B_{i}$
i=1	0.3	0.15	0.748	0.0013	0.72
i=2	0.19	0.095	0.8020	0.0043	0.83
i=3	0.14	0.07	0.792	0.0023	0.95
i=4	0.15	0.075	0.691	0.0015	1.88
i=5	0.04	0.02	0.2562	0.00012	0.83

**Table 2:** mD-H parameters for the 5DoF robotic arm.

Joints	$\theta_{i}$	$d_{i}$	$\alpha_{i-1}$	$a_{i-1}$
One	$\theta_{1}$	0	0	0
Two	$\theta_{2}$	0	$\frac{\pi }{2}$	$l_{2}$
Three	$\theta_{3}$	0	0	$l_{3}$
Four	$\theta_{4}$	0	0	$l_{4}$
Five	$\theta_{5}$	0	$-\frac{\pi }{2}$	$l_{5}$

**Table 3:** Numerical analysis for the $f_{obj}$ of GA, PSO and GSO for various sets of parameters.

	Runs	${\mathrm{set}}_{\mathrm{ga}}^{1}$	${\mathrm{set}}_{\mathrm{ga}}^{2}$	${\mathrm{set}}_{\mathrm{ga}}^{3}$
GA	1	4.33 $\times \;10^{-5}$	1.87 $\times \;10^{-5}$	6.12 $\times \;10^{-7}$
	2	0.0013	4.43 $\times \;10^{-5}$	1.6 $\times \;10^{-5}$
	3	1.24 $\times \;10^{-5}$	1.99 $\times \;10^{-4}$	5.23 $\times \;10^{-8}$
	4	1.7 $\times \;10^{-5}$	2.05 $\times \;10^{-5}$	2.96 $\times \;10^{-7}$
	5	4.25 $\times \;10^{-5}$	2.0 $\times \;10^{-5}$	1.76 $\times \;10^{-5}$
	6	2.43 $\times \;10^{-5}$	6.38 $\times \;10^{-4}$	3.6 $\times \;10^{-5}$
	7	3.913 $\times \;10^{-5}$	8.19 $\times \;10^{-5}$	7.5 $\times \;10^{-5}$
	8	3.74 $\times \;10^{-5}$	6.27 $\times \;10^{-5}$	6.65 $\times \;10^{-5}$
	9	3.95 $\times \;10^{-5}$	3.95 $\times \;10^{-5}$	1.75 $\times \;10^{-6}$
	10	1.13 $\times \;10^{-5}$	1.13 $\times \;10^{-5}$	6.96 $\times \;10^{-5}$
Mean		1.54 $\times \;10^{-4}$	3.83 $\times \;10^{-5}$	2.83 $\times \;10^{-5}$
Max		1.3 $\times \;10^{-3}$	8.19 $\times \;10^{-5}$	7.5 $\times \;10^{-5}$
variance		1.61 $\times \;10^{-7}$	5.87 $\times \;10^{-10}$	9.69 $\times \;10^{-10}$
H-value			0.03	
	**Runs**	${\mathrm{set}}_{\mathrm{pso}}^{1}$	${\mathrm{set}}_{\mathrm{pso}}^{2}$	${\mathrm{set}}_{\mathrm{pso}}^{3}$
PSO	1	1.14 $\times \;10^{-6}$	1.45 $\times \;10^{-11}$	6.20 $\times \;10^{-17}$
	2	5.43 $\times \;10^{-8}$	2.2 $\times \;10^{-6}$	9.41 $\times \;10^{-14}$
	3	0.14 $\times \;10^{-3}$	6.79 $\times \;10^{-17}$	6.79 $\times \;10^{-17}$
	4	8.57 $\times \;10^{-5}$	2.44 $\times \;10^{-14}$	1.99 $\times \;10^{-14}$
	5	2.63 $\times \;10^{-3}$	6.2 $\times \;10^{-17}$	6.2 $\times \;10^{-17}$
	6	2.5 $\times \;10^{-5}$	1.01 $\times \;10^{-11}$	6.2 $\times \;10^{-17}$
	7	3.33 $\times \;10^{-6}$	9.9 $\times \;10^{-12}$	1.0 $\times \;10^{-16}$
	8	7.28 $\times \;10^{-13}$	6.24 $\times \;10^{-10}$	5.66 $\times \;10^{-13}$
	9	3.79 $\times \;10^{-7}$	5.42 $\times \;10^{-16}$	6.2 $\times \;10^{-17}$
	10	3.68 $\times \;10^{-8}$	2.81 $\times \;10^{-11}$	6.2 $\times \;10^{-17}$
Mean		2.89 $\times \;10^{-4}$	2.2 $\times \;10^{-7}$	6.8 $\times \;10^{-14}$
Max		2.63 $\times \;10^{-3}$	2.2 $\times \;10^{-6}$	5.66 $\times \;10^{-13}$
Variance		6.79 $\times \;10^{-7}$	4.83 $\times \;10^{-13}$	3.14 $\times \;10^{-26}$
H-value			16.07	
	**Runs**	${\mathrm{set}}_{\mathrm{gso}}^{1}$	${\mathrm{set}}_{\mathrm{gso}}^{2}$	${\mathrm{set}}_{\mathrm{gso}}^{3}$
GSO	1	8.34 $\times \;10^{-8}$	6.2 $\times \;10^{-17}$	6.2 $\times \;10^{-17}$
	2	1.74 $\times \;10^{-7}$	3.03 $\times \;10^{-13}$	2.37 $\times \;10^{-16}$
	3	2.7 $\times \;10^{-13}$	2.45 $\times \;10^{-11}$	1.54 $\times \;10^{-15}$
	4	1.71 $\times \;\;10^{-5}$	1 $\times \;10^{-16}$	6.2 $\times \;10^{-17}$
	5	7.59 $\times \;10^{-11}$	7.85 $\times \;10^{-17}$	2.02 $\times \;10^{-16}$
	6	7.19 $\times \;10^{-6}$	6.79 $\times \;10^{-17}$	6.2 $\times \;10^{-17}$
	7	8.1 $\times \;10^{-4}$	5.43 $\times \;10^{-15}$	2.02 $\times \;10^{-16}$
	8	6.2 $\times \;10^{-5}$	7.76 $\times \;10^{-16}$	5.49 $\times \;10^{-17}$
	9	3.14 $\times \;10^{-7}$	6.2 $\times \;10^{-17}$	2.67 $\times \;10^{-14}$
	10	2.87 $\times \;10^{-12}$	7.63 $\times \;10^{-14}$	4.99 $\times \;10^{-14}$
Mean		9.97 $\times \;10^{-5}$	2.94 $\times \;10^{-12}$	7.9 $\times \;10^{-15}$
Max		8.1 $\times \;10^{-4}$	2.45 $\times \;10^{-11}$	4.99 $\times \;10^{-14}$
Variance		7.13 $\times \;10^{-8}$	5.98 $\times \;10^{-23}$	2.86 $\times \;10^{-28}$
H-value			15.84	

**Table 4:** Computational time for GA, PSO and GSO in seconds.

Runs	GA	PSO	GSO
1	8.35 (s)	2.46 (s)	3.87 (s)
2	8.18 (s)	2.41 (s)	3.81 (s)
3	8.01 (s)	2.35 (s)	3.83 (s)
4	8.11 (s)	2.40 (s)	3.77 (s)
5	8.09(s)	2.41 (s)	3.45 (s)
6	8.33 (s)	2.36 (s)	3.80 (s)
7	8.26 (s)	2.39 (s)	3.86 (s)
8	8.26 (s)	2.35 (s)	3.54 (s)
9	8.13 (s)	2.37 (s)	3.88 (s)
10	8.38 (s)	2.43 (s)	3.84 (s)
Mean	8.21 (s)	2.39 (s)	3.76 (s)

**Table 5:** Coordinates and angles of the target points.

Positions		Angles			
**Points**	**Coordinates**	θ	$\theta_{2}$	$\theta_{3}$	$\theta_{4}$
A	(0.11,0.25,0.14)	2.41	1.26	1.705	−0.83
B	(0.21,0.32,0.22)	2.35	1.37	0.63	−0.029
C	(0.12,0.14,0.12)	2.11	1.17	1.21	0.75
D	(0.19,0.14,0.05)	2.08	1.58	1.36	−0.95
E	(0.19,−0.1,0.5)	1.25	0.3	0.59	1.04
F	(0.21,−0.16,0.7)	1.13	0.72	0.07	−1.17
G	(0.15,0.1,0.3)	1.94	0.45	1.35	0.93
H	(0.14,−0.11,0.14)	1.16	1.49	0.34	1.67
I	(0.12,0.15,0.05)	2.15	1.44	1.49	−0.17

**Table 6:** PID parameters tuned by PSO.

	PSO			GA			GSO		
	$K_{p}$	$K_{i}$	$K_{d}$	$K_{p}$	$K_{i}$	$K_{d}$	$K_{p}$	$K_{i}$	$K_{d}$
Joint 1	15.35	34.3233	2.8305	51.2684	175.3676	0.2106	36.2147	295.5165	0.2556
Joint 2	26.8257	25.2366	7.8637	59.9833	173.4063	8.6907	39.5278	242.1184	8.4769
Joint 3	13.3082	15.4017	3.3305	76.2230	160.6402	3.0397	83.4758	175.8795	3.3379
Joint 4	5.4971	6.4876	3.4602	83.6052	189.7420	7.7188	68.0942	183.7764	4.1200

**Table 7:** Angular trajectory average error for optimal tuned controllers.

	${\mathrm{AE}}_{\mathrm{GA}}$	${\mathrm{AE}}_{\mathrm{PSO}}$	${\mathrm{AE}}_{\mathrm{GSO}}$
Joint 1	0.0561	0.05871	0.5407
Joint 2	0.0348	0.0313	0.0304
Joint 3	0.0728	0.0794	0.0675
Joint 4	0.0621	0.0605	0.0488

## Data Availability

Not applicable.
